# Bone Mineral Density in Postmenopausal Women Heterozygous for the C282Y HFE Mutation

**DOI:** 10.1155/2016/5638273

**Published:** 2016-03-31

**Authors:** Jenny E. Gunton, Frances Gates, Greg R. Fulcher, Phillip B. Clifton-Bligh

**Affiliations:** ^1^Westmead Clinical School, Westmead Hospital, Cnr Hawkesbury Road and Darcy Road, Westmead, NSW 2145, Australia; ^2^Department of Endocrinology, Royal North Shore Hospital, St Leonards, NSW 2065, Australia; ^3^Northern Clinical School, University of Sydney, Royal North Shore Hospital, St Leonards, NSW 2065, Australia

## Abstract

Mutations in the HFE gene may be associated with increased tissue iron stores reflected in an elevated serum ferritin. With homozygous mutation C282Y, the increase in serum ferritin may be associated with tissue damage in the liver, pancreas, and pituitary and with a reduced bone mineral density. With heterozygous mutation C282Y, the degree of iron retention is less but information relating to how a heterozygous C282Y mutation might impact bone mineral density is uncertain. The present study was undertaken to study the relationships between bone mineral density measured by dual energy X-ray absorptiometry and the serum ferritin and serum iron in postmenopausal women heterozygous for the C282Y mutation. The spinal bone mineral density, L2–4, was significantly less than age matched community controls (*P* = 0.016). There was no significant change in the femoral neck bone mineral density compared to age matched community controls. The correlation between the spinal bone mineral density, L2–4, the femoral neck bone mineral density, and the serum ferritin was not significant. The serum iron correlated significantly inversely with the femoral neck bone mineral density (*P* = 0.048). The heterozygous C282Y mutation may be associated with impairment of bone cell function in postmenopausal women when only small increases in the serum iron or serum ferritin have occurred.

## 1. Introduction

Osteoporosis associated with hemochromatosis in men was first described by Delbarre in 1960 [1] and confirmed by Pawlotsky et al. in 1979 [2]. Hemochromatosis due to excess tissue iron storage is frequently due to a homozygous mutation, cysteine to tyrosine, C282Y, in the HFE gene. The prevalence of osteoporosis in hemochromatosis and the pathogenic mechanisms involved are not completely understood, for example, whether there is a relationship between the serum ferritin and bone mineral density.

In men with hemochromatosis and a serum ferritin between 350 and 8410 *μ*g/L, the presence of osteoporosis was associated with a lower serum free testosterone [3]. However, in men with the homozygous HFE mutation, the serum ferritin and the serum testosterone did not differ between those with and without osteoporosis. Most osteoporotic men were not hypogonadal (76.9%) [4]. Interestingly, in men heterozygous for the HFE mutation, C282Y, but without iron overload, the serum SHBG was elevated but the serum testosterone was normal [5].

Significant iron overload may cause chronic liver disease. Liver disease was associated with a lower bone mineral density in men of all ages and in women older than 60 years [6]. In a three-year longitudinal study of postmenopausal women, higher serum ferritin levels were correlated with increased bone loss at the femoral neck [7]. The mean serum ferritin in these women was 76.9 ± 50.6*μ*g/L. However, in postmenopausal women a higher serum ferritin was not correlated with a lower bone mass [8, 9]. In postmenopausal women with and without osteoporosis the serum ferritin was not significantly different between the two groups [10], but in another study of postmenopausal women, mean age 73 years, who had sustained a hip fracture, those who had a serum ferritin above 150 *μ*g/L had a lower bone density than those in whom the serum ferritin was less than 150 *μ*g/L [11].

In a study that looked at transiliac biopsy samples the concentration of iron in the cortical bone was greater in osteoporotic participants than in nonosteoporotic participants (*P* ≤ 0.01) [12]. In a rat study, ovariectomy was associated with an increased concentration of iron in bone and the loss of bone mass and the deterioration of bone microarchitecture was ameliorated by oral treatment with an iron chelator [13]. In postmenopausal women, the serum ferritin was higher, 71 *μ*g/L, than in premenopausal women, 37 *μ*g/L. The postmenopausal estradiol levels were not correlated with the serum ferritin [14].

A question arises as to what happens to the serum ferritin in postmenopausal women with the heterozygous C282Y mutation in the HFE gene and whether the serum ferritin is related to bone mineral density in this group of women. The serum ferritin levels were not different between those heterozygous for the C282Y HFE mutation and those without the mutation between the ages of 20 and 70. In women aged 50–79 the geometric mean for the serum ferritin was 87 *μ*g/L [15]. In postmenopausal women aged 54–64 there was a slight increase in the serum ferritin in women heterozygous for the HFE mutation, 113.5 *μ*g/L (median), compared to 101.0 *μ*g/L (median) in those without the mutation [16].

In another study in women aged 61–90 with the heterozygous C282Y HFE mutation the serum ferritin was significantly higher than in women without the mutation (geometric mean 79 *μ*g/L compared to 48 *μ*g/L). The 95% confidence interval for serum ferritin in women with the heterozygous C282Y HFE mutation was 61–98 *μ*g/L [17]. Relatively small increases in the serum ferritin in postmenopausal women may be associated with an increased rate of loss of bone mineral density [7]. With this in mind we studied bone mineral density in postmenopausal women heterozygous for the C282Y HFE mutation.

## 2. Methods

All postmenopausal women in our institution who had had an HFE gene analysis between 1999 and 2012 were identified. Women homozygous for the C282Y mutation and women who were either homozygous or heterozygous for the H63D (His → Asp at residue 63) mutation were excluded. Women with celiac disease, hyperparathyroidism, or hyperthyroidism were also excluded. At the same time, the serum iron and serum ferritin were measured and liver function tests were carried out. Between 1999 and 23 March 2001, the serum ferritin was measured by the Roche immunoturbidimetric assay method (reference range 15–150 *μ*g/L), and between 23 March 2001 and 26 October 2012, Roche E170 chemiluminescent assay was used (reference range 15–400 *μ*g/L). Between 1999 and 23 March 2001 the serum iron was measured by Roche Hitachi 747 method (reference range 8–30 *μ*m/L) and between 23 March 2001 and 26 October 2012 Roche Modular was employed (reference range 8–30 *μ*m/L).

Of the 137 postmenopausal women who were heterozygous for C282Y, 26 also had bone density measurements of the lumbar spine (L2–L4) and femoral neck. The measurements were made on a Norland XR26 bone densitometer and converted to Hologic equivalents using the formula of Genant et al. [18]. Using the data from the National Health and Nutritional Examination Survey 2005–2008 for white non-Hispanic women [19], *z*-scores for the lumbar spine and femoral neck bone densities were calculated to determine if the age matched bone density measurements (*z*-scores) were significantly different on average from the general population. Bone mineral density measurements in Australian women were previously found to be very similar to those of women in the USA using NHANES data [20]. The relationship between the serum iron and serum ferritin and bone mineral density at the lumber spine and femoral neck was also studied. The pathology testing was supported by the hospital pathology laboratory (PaLMS).

## 3. Statistics and Ethics

Two-sided *t*-tests were used to compare *z*-scores with *z*-scores for the general population. The Pearson correlation test was used to study the relationship between the serum iron and serum ferritin and bone mineral density. A *P* value of <0.05 was considered statistically significant. Unless otherwise specified, the data are presented as mean ± standard deviation (SD). The study was approved by the Northern Sydney Local Health District Human Research Ethics Committee (NSLHD HREC). Because this was a retrospective study of data already accumulated by treating clinicians as part of general medical care, informed consent from individual participants was not required.

## 4. Results

The age of the study participants ranged between 48 and 81 years (average 62.3 ± 9.3 SD). Women over the age of 60 or in whom one year had elapsed since the last period were assumed to be menopausal. Those under the age of 60 not receiving oestrogen replacement had serum oestradiol levels of 38 pmol/L, 39 pmol/L, and 45 pmol/L, within the menopausal range. All women were white Caucasian. Of the 26 women, 6 were currently receiving estrogen therapy, 3 had previously received estrogen therapy, and 8 were currently on bisphosphonate therapy (alendronate, risedronate, or etidronate). One participant had received regular venesection because of raised serum iron and raised iron binding saturation. Three were receiving thyroxine therapy and had normal thyroid function tests. Three women with significant liver function abnormality and two who were currently receiving prednisone were excluded from the study. Finally 21 women were included in the analysis.

The mean serum ferritin for the group was 220.1 ± 129.8*μ*g/L. The mean serum iron was 17.3 ± 7.5*μ*m/L. The *z*-scores for bone density at the lumbar spine and femoral neck are shown in Table 1.

The average *z*-score for the lumbar spine, L2–L4, was −0.44 ± 0.77 and was significantly different from 0 (*P* = 0.016). The average *z*-score for the femoral neck was −0.19 ± 0.69, not significantly different from 0 (*P* = 0.221) (Tables 2 and 3).

The correlation coefficients relating the serum ferritin to the bone mineral density of the lumbar spine, L2–L4, and femoral neck were not significant for the lumbar spine (*r* = 0.019, *P* = 0.936) or for the femoral neck (*r* = −0.093, *P* = 0.688) (Table 4).

The correlation coefficients relating the serum iron to the bone mineral density of the lumbar spine and femoral neck were not significant for the lumbar spine (*r* = −0.236, *P* = 0.303) but were significant for the femoral neck (*r* = −0.436, *P* = 0.048) (Table 4).

In this study, women receiving estrogen replacement or bisphosphonate therapy were included in the analysis. Therapy with either of these agents would have a tendency to increase bone mineral density so that any reduction in bone density is likely to have been greater before therapy.

## 5. Discussion

The link between excess tissue iron stores and osteoporosis was first noted by Delbarre in 1960. Excess tissue iron accumulation as reflected in an elevated serum ferritin is frequently seen in persons homozygous for the C282Y HFE mutation and many of these patients may develop osteoporosis [21]. Additional risk factors for the development of osteoporosis are hypogonadism in men and chronic liver disease in men and women [21].

The question arises as to whether milder degrees of tissue iron storage are associated with reduced bone density. In a group of postmenopausal women, the serum ferritin in those with osteoporosis was not different from those who were not osteoporotic [10]. Also in postmenopausal women a higher serum ferritin was not correlated with a lower bone mass [8, 9]. However, in a longitudinal study over 3 years in postmenopausal women, the serum ferritin was correlated with an increased loss of bone mineral density in the femoral neck with serum ferritin levels within the normal range [7], and in another study of postmenopausal women, mean age 73 years, who had sustained a hip fracture, those with a serum ferritin above 150 *μ*g/L had a lower bone density than those in whom the serum ferritin was less than 150 *μ*g/L [11].

In the present study of postmenopausal women heterozygous for the C282Y HFE mutation, the spinal bone mineral density of L2–L4 was significantly less than that of the average population mean corrected for age. The femoral neck *z*-scores for bone mineral density were not significantly lower than the average population mean. The serum ferritin was not correlated with the bone mineral density at the lumber spine or the femoral neck, a finding similar to that found in postmenopausal women studied by Lee et al. in whom the average serum ferritin was 77.1 *μ*g/L [9]. The average serum ferritin in the present study was 220.1 *μ*g/L, considerably higher than that found in the study of Kim et al. [7] and higher than average serum ferritin levels of 64 *μ*g/L found in women with average age 52.8 years heterozygous for the C282Y HFE mutation in the study of Rossi et al. [15].

In another study of women aged 61–90 with the heterozygous C282Y HFE mutation the serum ferritin was 79 *μ*g/L (geometric mean) [17]. In a longitudinal study of C282Y HFE simple heterozygotes followed over 12 years the serum ferritin levels of postmenopausal women did not change significantly and were not significantly different compared to the serum ferritin levels of postmenopausal women without the C282Y HFE mutation [22].

In a population study of Australian women greater than 50 years the median serum ferritin was 80.5 *μ*g/L [23]. A further study of postmenopausal women with osteoporosis found no difference in the serum ferritin between those with and without osteoporosis but the serum iron was slightly lower in those with osteoporosis (*P* = 0.047). This was linked to a variation in the haptoglobin molecule which binds heme more avidly and reduces the amount of free iron available to tissues and which protected against fracture in postmenopausal women with osteoporosis [24].

Excess tissue iron has been implicated in impairment of bone metabolism. In the zebra fish exposure to ferric iron increased the expression of TRACP-5b of osteoclast origin and treatment with deferoxamine increased bone mineralization [25]. In mice homozygous for the C282Y HFE mutation excess iron was seen on the surfaces of bone trabeculae and there was a decrease in the number and thickness of trabeculae. There was no change in osteoblast surface but osteoclast numbers were increased [26]. In a rat study, ovariectomy was associated with an increased concentration of iron in bone and the loss of bone mass and the deterioration of bone microarchitecture was ameliorated by oral treatment with an iron chelator. Bone resorption was reduced. The serum ferritin was not measured in this study [13].

In a study which looked at transiliac bone biopsy samples, the concentration of iron in cortical bone was greater in osteoporotic participants than in nonosteoporotic participants [12]. Hepcidin of hepatic origin influences gastrointestinal iron absorption. Increased levels of serum hepcidin decrease iron absorption and low levels increase iron absorption. BMP-2 increased hepcidin mRNA expression in liver cells and this was enhanced by hemojuvelin which acts as a coreceptor for BMP-2 [27] and which was independent of the effect of HFE [28]. Higher serum ferritin levels were associated with particular SNPs in the BMP-2 gene [29]. BMP-2 has a role in the differentiation and function of osteoblasts in bone so that diminished function may reduce bone mass and diminish hepatic hepcidin secretion. Iron absorption may increase as a result. Serum hepcidin was not measured in the present study.

In a mouse study using C2C12 preosteoblasts in culture, increased iron and ferritin in the cell culture media in combination with low estradiol levels inhibited the effect of BMP-2 on cell differentiation. Also mice heterozygous for the HFE mutation had lower femoral bone mineral density compared to wild type mice [30].

The present study is the first to examine bone mineral density in postmenopausal women heterozygous for the C282Y HFE mutation which showed a significant reduction in the lumbar spine bone mineral density when compared to age matched community controls. However, there are several important limitations in this study which must be regarded only as exploratory. Firstly, the C282Y HFE heterozygotes were identified from an already established data base and it is not clear why in individual cases the gene analysis was done in the first place. Because the mean serum ferritin was 220.1 *μ*g/L, higher than the average values expected in postmenopausal women, it is likely that the HFE mutation was sought following the finding of a higher than average serum ferritin. Also it is likely that the HFE analysis was carried out in relatives of known C282Y HFE homozygotes. If the HFE mutation was sought specifically in postmenopausal women with known low bone mineral density then this would introduce substantial bias.

Women receiving estrogen or bisphosphonates were included in the analysis even though these medications were likely to have increased the bone mineral density and make it less likely to detect a significant reduction in age matched bone mineral density. Even so, despite the inclusion of these participants, in this small number of 21 postmenopausal women there was a highly significant (*P* = 0.016) reduction in the L2–L4 bone mineral density compared to age matched controls, strengthening the concept that C282Y HFE heterozygosity is associated with reduced bone mineral density in the lumbar spine.

Nevertheless, in order to confirm or refute the results of the present study, postmenopausal women selected randomly from the population at large should have measures of bone mineral density, serum iron, and serum ferritin and also studies for the heterozygous C282Y HFE mutation so that unbiased comparisons could be made between those with and those without the mutation.

It is not known if there is a threshold for serum ferritin above which there is an impairment of bone formation or an increase in bone resorption or if C282Y HFE heterozygotes who are osteoporotic respond to therapy designed to increase bone mineral density in the same way as persons without the mutation. Although venesection of one premenopausal woman with an initial serum ferritin of 513 *μ*g/L in a separate study was associated with a significant increase in the bone mineral density of the lumbar spine and the femoral neck after one year [31], additional studies are required to determine at what levels of serum ferritin venesection may be helpful in increasing bone mineral density in postmenopausal women with osteoporosis and mild increases in the serum ferritin. In the present study, the serum ferritin in C282Y HFE heterozygotes ranged between 12 and 543 *μ*g/L. Three patients were excluded from analysis because abnormal liver function tests had serum ferritin levels of 540, 595, and 634 *μ*g/L.

In conclusion, in the present study, C282Y HFE heterozygous postmenopausal women were shown to have significantly lower bone mineral density of the lumber spine, L2–L4, compared to age matched community controls. In this small study the serum ferritin was not correlated with measures of bone mineral density in the spine or femoral neck.

## Figures and Tables

**Table 1:** Postmenopausal women heterozygous for the C282Y HFE mutation bone mineral density *z* scores and serum ferritin and serum iron.

	L2–4	FN	Ferritin	Iron
(1)	−0.95	−0.06	155	12
(2)	−0.27	+0.10	155	11
(3)	−0.68	−0.03	241	17
(4)	+0.56	+0.01	331	16
(5)	+0.93	−0.24	148	12
(6)	−0.91	−0.81	196	12
(7)	+0.16	−0.61	234	26
(8)	−0.94	−0.30	166	33
(9)	−0.86	+0.76	417	12
(10)	−0.12	−0.91	543	30
(11)	−1.02	−0.51	12	12
(12)	−0.93	−0.55	220	31
(13)	−1.70	+0.39	240	16
(14)	−0.41	−0.63	274	16
(15)	−0.30	+0.86	92	12
(16)	+0.10	+0.84	411	8
(17)	+0.28	+0.86	28	14
(18)	−1.50	−1.38	177	17
(19)	−1.06	−1.46	312	17
(20)	+1.23	+0.16	135	10
(21)	−0.89	−0.46	66	29

Serum Ferritin *µ*g/L.

Serum Iron *µ*mol/L.

L2–4: BMD of lumbar spine.

FN: BMD of femoral neck.

**Table 2:** Mean value for bone mineral density *z*-scores for lumbar spine and femoral neck.

	Mean	Standard deviation
L2–L4 *z*-score	−0.44	0.77
FN *z*-score	−0.19	0.69

**Table 3:** *t*-test of whether *z*-scores for bone mineral density significantly differ from 0.

	Test value = 0					
	*t*	df	*P* value (2-tailed)	Mean difference	95% confidence interval of the difference	
					Lower	Upper
L2–L4 *z*-score	−2.633	20	0.016	−0.44	−0.79	−0.09
FN *z*-score	−1.263	20	0.221	−0.19	−0.50	0.12

**Table 4:** Pearson correlations between serum ferritin, serum iron, and *z*-scores.

	L2–L4 *z*-score	FN *z*-score
Pearson correlation	*r* = 0.019	*r* = −0.093
Serum ferritin *P* value (2-tailed)	*P* = 0.936	*P* = 0.688
Pearson correlation	*r* = −0.236	*r* = −0.436
Serum iron *P* value (2-tailed)	*P* = 0.303	*P* = 0.048
